# Functional Networking of Human Divergently Paired Genes (DPGs)

**DOI:** 10.1371/journal.pone.0078896

**Published:** 2013-10-31

**Authors:** Bin Xie, Dapeng Wang, Yong Duan, Jun Yu, Hongxing Lei

**Affiliations:** 1 CAS Key Laboratory of Genome Sciences and Information, Beijing Institute of Genomics, Chinese Academy of Sciences, Beijing, China; 2 University of Chinese Academy of Sciences, Beijing, China; 3 UC Davis Genome Center and Department of Biomedical Engineering, Davis, California, United States of America; Ecole Normale Supérieure de Lyon, France

## Abstract

Divergently paired genes (DPGs), also known as bidirectional (head-to-head positioned) genes, are conserved across species and lineages, and thus deemed to be exceptional in genomic organization and functional regulation. Despite previous investigations on the features of their conservation and gene organization, the functional relationship among DPGs in a given species and lineage has not been thoroughly clarified. Here we report a network-based comprehensive analysis on human DPGs and our results indicate that the two members of the DPGs tend to participate in different biological processes while enforcing related functions as modules. Comparing to randomly paired genes as a control, the DPG pairs have a tendency to be clustered in similar “cellular components” and involved in similar “molecular functions”. The functional network bridged by DPGs consists of three major modules. The largest module includes many house-keeping genes involved in core cellular activities. This module also shows low variation in expression in both CNS (central nervous system) and non-CNS tissues. Based on analyses of disease transcriptome data, we further suggest that this particular module may play crucial roles in HIV infection and its disease mechanism.

## Introduction

Divergently paired genes (DPGs) are also known as bi-directionally expressed and head-to-head oriented genes, which can be further defined based on their TSS (transcription start site) distances, such as within or beyond 1 kb between the gene pairs [1]. DPGs accounted for ∼10% of all human genes [1], [2] and may have distinct functional relevance when analyzed in a context of interaction networks. As a highly conserved gene organization, the two members of DPGs are most likely to share the same promoters and play unique roles that differentiate them from other forms of gene organization, such as head-to-tail, tail-to-tail, ncRNA-proteic pair and random pairs [3].

Several investigations have been conducted on DPGs in recent years, especially on those of human [1], [2], [4], [5] and Drosophila [6], [7]; most of them are focused on the sequence signatures, structural features and functional elements of DPG promoter regions. For instance, enrichment of certain motifs [8], predominance of CpG-islands [9] and exclusion of nucleosomes [10]. Many of these DPG studies have been focused on conserved sequence patterns of the paired genes, including microsynteny across metazoan DPGs [11] and TSS distance across diverse species [12]. We have also pointed out that the conservation of DPGs may be very different for arthropods and vertebrates [7]. However, the reasons and mechanisms why DPGs are conserved and how they are involved in functional networks remain to be elucidated in details although the functional relevance has been pointed out repeatedly based on co-expression and basic gene functional annotation [13]. For instance, it has been proposed that DPGs are paired for similar functions (such as DNA repair) and often highly correlated in expression [14], [15]. Therefore, investigation on functional connections of DPGs both within the paired genes and among the pairs becomes necessary.

Here we report an examination of human DPGs and the differences between DPGs and random gene pairs in their functional connections and regulatory roles in normal tissues and disease processes. Functional similarity of DPGs is tested based on Gene Ontology (GO, http://www.geneontology.org/) annotations and regulatory roles are examined based on functional interactions or networking linked by the DPG pairs. We also compare DPG gene expression patterns in 65 normal human tissues and several common disease samples. We use HIV as an example to illustrate the role of DPGs played in immune defense mechanisms.

## Results and Discussion

### An overview of DPGs

There are 1,063 pairs of human DPGs recorded in the LCGbase [16], slightly less than what has been reported in previous studies, ranging from 1,262 to 1,446 pairs [1], [7], [17]. Considering the information on gene ID transformation from both NCBI and HGNC databases, we focused our analysis on 864 pairs of DPGs (1,728 genes), among which 682 have positive TSS distance (0 kb < TSS distance <1 kb) and the remaining 182 pairs have negative TSS distance (−1 kb < TSS distance <0 kb; **Table S1**). When considering both gene density and chromosome length, we found that DPGs were enriched on chromosomes 1 and 2. We also found that DPGs was absent on chromosome Y, which might be due to the fact that the small number of genes still present on it. Chromosome Y is known to be fast evolving, and it poses great challenge to preserve the genomic structure of DPGs on this chromosome [18], [19]. In particular, DPGs are under negative selection to maintain their relative position relations to be prevented from the neutral mutation of separating them in the process of relatively large segmental sequence variation events of the chromosome evolution.

Next, we conducted GO term-based functional enrichment analysis on 1,728 DPGs using hypergeometric test. On the one hand, consistent with previous studies [4], [6], [7], [20], the overrepresented functions include RNA process, DNA repair and cell cycle, which are, by and large, primary cellular functions that are shared by all eukaryotes, even unicellular ones. On the other hand, the underrepresented functions include signal transduction, immune response, and development process, which are secondary cellular functions shared by animals. More specifically, DPGs on chromosome 1 tend to function in DNA packaging and ncRNA metabolic process, and similarly nucleosome assembly on chromosome 6, translation on chromosome 9, cell cycle on chromosome 11, protein folding on chromosome 14, mRNA processing on chromosome 19, and RNA splicing on chromosome 22.

### Functional divergence of DPG pairs

**Figure 1:**
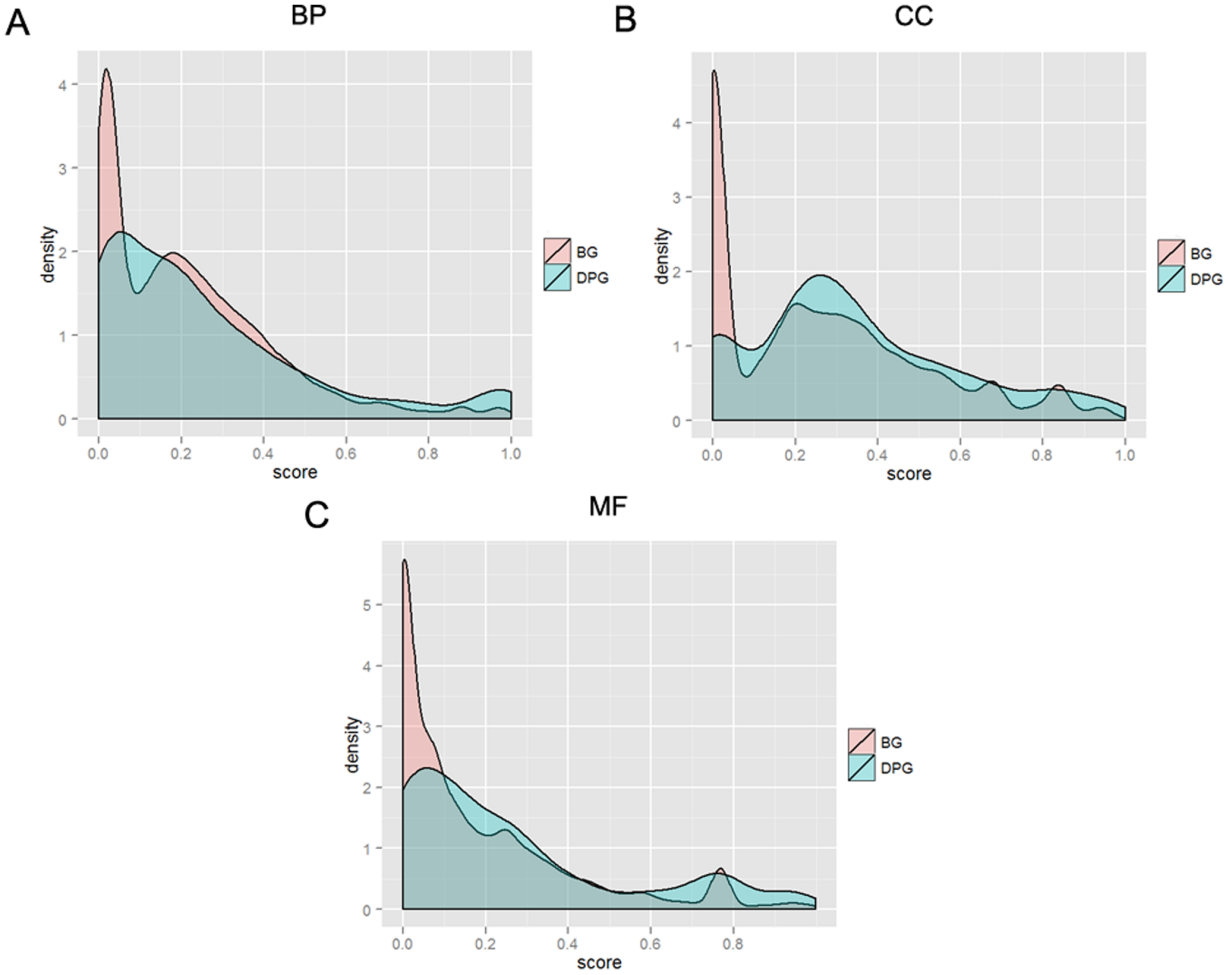
Comparison of GO similarity between DPGs and random gene pairs. Frequence distributions of GO similarity score on biological process (BP, A), cell component (CC, B), and molecular function (MF, C). The blue and red lines depict frequency distributions of DPGs and the randomly sampled gene pairs, respectively.

As physically neighbouring pairs, the functional similarity within a DPG is of great interest. We calculated the GO similarity score of gene pairs of DPGs (details in Methods) and compared the distribution of these scores to random pairing using Kolmogorov-Simirnov test. Our main findings are four folds. First, DPGs are not significantly different from random gene pairs in biological process (BP; p value = 0.06234) but significantly different in cell component (CC; p value = 7.44E-15) and molecular function (MF; p value  = 1.73E-11). In details, we observed the left shift of the distribution line of BP and the right shift of those of CC and MF, in comparison to the background (BG; **Figure 1**). Second, gene pairs with paralogs in DPGs somewhat influence the result of this similarity analysis. We can see a peak close to score 1.0 on the map of BP but the DPG pairs attributed to this peak are largely 12 pairs of histone genes. If we exclude these histone pairs and then compare to the background, the p value becomes 0.2559. We also found that these 12 gene pairs form nearly independent modules in DPG functional networking. Third, DPGs have higher tendency to be paired for similar cellular components. In addition to the smaller p value and the rightward shift of the distribution, we also found that the right peak is much higher than the left one on the map of CC, contrary to the background. Fourth, there is no significant correlation between TSS distance of DPGs and functional similarity, regardless of BP, CC, or MF.

Our analysis indicates that DPG pairs are not formed randomly as the two genes within a DPG pair tend to be involved in similar “cellular component” and “molecular function” but become divergent in “biological process”. We therefore propose that the genomic arrangement of DPGs facilitate the regulation of two biological functions that might be related. For example, none of the 134 DPGs involved in RNA process fells in the same gene pair, and the functions of the paired genes are mainly related to protein localization, phosphorylation and transcription, and preferably associated with mitochondrion, ribosome, and nucleolus. Only two of the 73 DPGs involved in DNA repair formed a pair (SMC6 and GEN1) and the functions of the paired genes are enriched in energy metabolism, RNA processing, and protein localization, and again preferably associated with mitochondrion, ribosome, and nucleolus. In addition, this observation is further supported when we examine individual DPG pairs and their functional networking.

**Figure 2:**
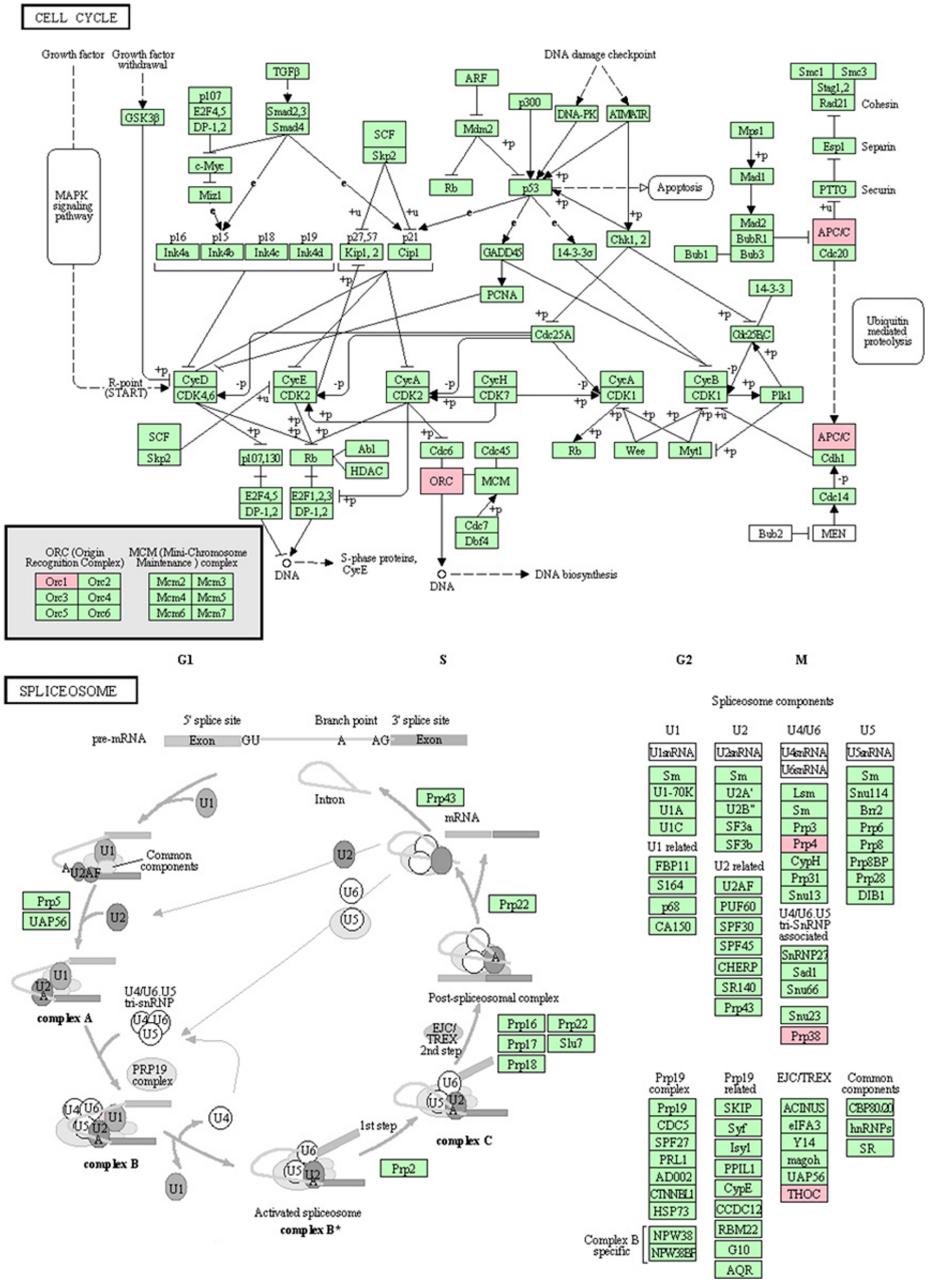
The distribution of three DPGs in two KEGG pathways: cell cycle and splicesome. Three DPGs are 4998 (ORC1)-84950 (PRPF38A), 246184 (CDC26)-9128(PRPF4), and 10189 (ALYREF)-51529 (APC11). These six genes act as bridges in the regulation between DNA replication, proteolysis, RNA splicing, and cell cycle; the figure is drawn based on KEGG pathway mapping tool.

So far, we had 419 out of 1,728 human DPGs annotated in KEGG (Kyoto Encyclopedia of Genes and Genomes), among which 120 DPGs formed 60 pairs. If excluding 12 pairs of histone genes and 6 pairs of gene families, we only had 2 DPGs or 4 genes involved in the same KEGG pathway; PPAT and PAICS are enzymes that regulate the step 1 and step 6/7 of *de novo* purine nucleotide biosynthetic pathway, respectively; PRKDC and MCM4 participate in cell cycle, where PRKDC regulates DNA double-strand break repair and recombination, and MCM4 acts as a DNA unwinding enzyme and controls the initiation of eukaryotic genome replication. The rest of the DPG pairs annotated in KEGG are all involved in different pathways. Here we used “spliceosome” and “cell cycle” as examples to show the relationships between the gene pair of a DPG. Three DPGs were involved in the two pathways, including ORC1-PRPF38A, CDC26-PRPF4, and THOC4-APC11 (**Figure 2**). This suggests that splicing and cell cycle are two tightly linked processes through the regulation of three DPG pairs or six genes. More specifically, the co-regulation of ORC1 and PRPF38A functions in both initiation of DNA replication during cell cycle and U4/U5/U6 small nuclear RNAs binding that is directly involved in pre-mRNA splicing. Both CDC26 and APC11 are highly conserved components of the APC complex that functions as a cell cycle-regulated ubiquitin-protein ligase. Although only three DPG pairs are involved in the two pathways, their functions cover DNA replication, proteolysis, RNA splicing, and cell cycle.

One previous study by Li et al indicated that DPGs prefer similar biological process, but using only 267 annotated DPGs (21.15%) and Resnik's method [17], [21], and the authors also did not consider “shallow annotation problem” indicated by Sevilla et al [22] and the bias due to paralogous genes. Having examined 864 DPG pairs, we propose that DPGs do not have the tendency to share similar functions as previously claimed. Rather, DPGs have the tendency to reside on the same cellular component (not necessarily to have the same function) and regulate related (not the same) pathways.

### A functional network bridged by DPGs

To clearly demonstrate relationships among cellular functions connected by DPGs, we first constructed two network maps, an overlap map and an interaction map (**Figure S1 and S2**). The overlap map is based on the overlap of DPGs between a pair of functional gene sets, whereas the interaction map is based on the bridging of a pair of functional gene sets by a DPG gene pair. When the two maps are compared, only the DNA packaging and the cancer signaling modules show consistency. The DNA packaging module is mainly attributed to the histone gene pairs as described earlier, whereas the functional sets in the cancer signaling module are mostly small gene sets and connected by only one DPG pair. Other than the two modules, we observed that many cellular functions are coupled by DPG pairs despite the lack of connection on the overlap map.

**Figure 3:**
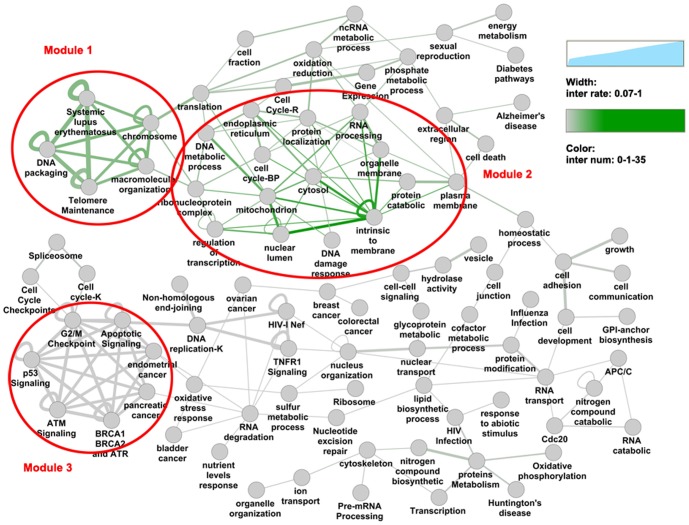
The interaction map of functional sets that linked by DPGs. We name the functional sets that linked by DPGs as interacted sets, and the linkage is based on the situation that one gene of a DPG pair is annotated for one function and the other for another functions. The interaction rate is related to the linked DPGs number and the set size. Interaction rate >7% is chosen to cover more functional sets. The edge color depends on the DPGs number that linked two adjacent sets, and the edge width depends on the interaction rate between two sets. Module 1 is related to histone and DNA packaging. Module 2 is related to regulation of cell cycle, gene expression, energy and some other functions. Module 3 is related to cancer signaling.

We organized the interaction map in a clear layout (**Figure 3**) by setting the interaction rate >0.07 to maximize the coverage of functional sets (90 functional sets, 1,262 DPGs, relative coverage 92.86%) and to demonstrate the functional connections. Since DPG gene pairs share the same promoter sequences, the adjacent nodes on the interaction map are most likely to have better correlated expression. Based on the pathway absolute score calculated from dataset GSE3526 (details in Methods), the top 10 correlated DPGs are highlighted on the interaction map (**Figure S3**), all of which reside in the densely connected regions.

To better understand the functional roles of DPGs, we used Cytoscape plugin “ClusterOne” and found 3 modules in the network [23]: (1) related to histone and DNA packaging (p value 0.005); (2) related to regulation of cell cycle, gene expression, energy, and some other functions (p value 4.908E-6); (3) related to cancer signaling (p value 9.923E-4; **Table S2**). Module 1, including 7 functional gene sets and 162 DPGs, is formed by 17 DPG pairs, among which 12 pairs are histone genes. Module 2 is connected by 303 DPG pairs and includes 15 functional gene sets and 1,068 DPGs. Module 3 is joined by only one DPG pair, and therefore is not included in further statistical analysis.

**Figure 4:**
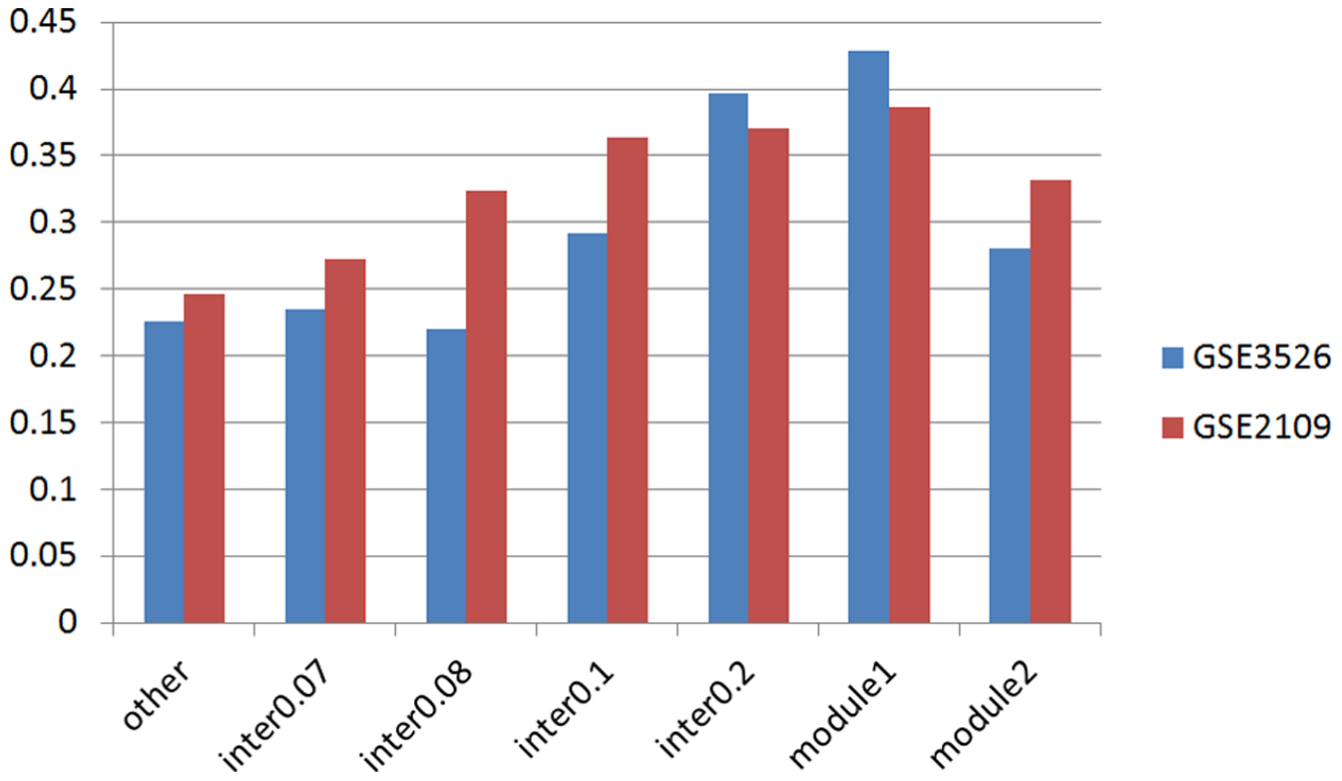
The average correlation value of DPGs. The average correlation value of DPGs is calculated between the pairs that linked two functional sets and without the linkage. The absolute correlation value is used, and the average correlation value is calculated based on the quartiles. Similar to Figure 3, the interaction rate >0.07 is first selected to form the interaction map, and then the DPGs which linked the sets are chosen to calculate the average correlation value, which is shown in this figure as “inter0.07”. “Other” indicates the value of other DPGs. “inter0.08”, “inter0.1”, and “inter0.2” shows the corresponding interaction rate >0.08, >0.1, and >0.2. “Module 1” and “Module 2” display the correlation value of DPGs that form module 1 and module 2. Correlation value is calculated based on GSE3526 and GSE2109.

We evaluated the average correlation value of DPGs within the two main modules. Similar to what's shown in Figure 3, the interaction rate >0.07 was first selected to construct the interaction map, and the DPGs that link the sets were chosen to calculate the average correlation value of gene expression, which was shown as “inter0.07” (**Figure 4**). The “other” set corresponded to the average value of all DPGs that were excluded by the cutoff. Three other sets, “inter0.08”, “inter0.1”, and “inter0.2”, corresponded to interaction rate >0.08, 0.1, and 0.2, respectively. “Module 1” and “module 2” related to the average correlation value of DPGs constituting module 1 and module 2, respectively. From the figure, we can find that the average correlation value of the connecting DPGs increased with the interaction rate. When we compared these modules, DPGs connecting the sets of module 1 have much higher correlation value than those of module 2. This indicates that the functions of module 2 are much more diverse than those of module 1.

### The distribution of HKGs (housekeeping genes) on the functional network

**Figure 5:**
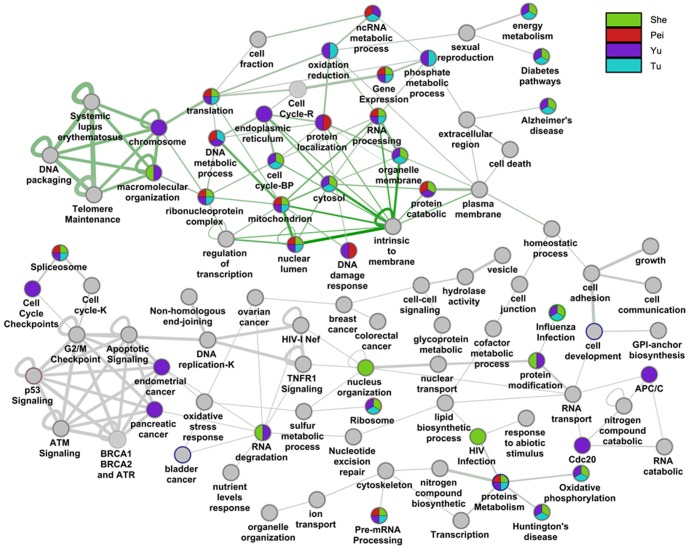
Cross comparison of House-keeping Genes (HKGs) functional categories with the DPG network. This figure shows the distribution of HKGs on the network connected by DPG pairs. The HKG lists are collected from three different studies.

Since DPGs and HKGs are both involved in the basic cellular functions, a thorough examination of their relationship becomes necessary at this point. We compared DPGs with a HKG list curated previously based on microarray-based gene expression profiling data [24], [25], [26], supplemented with a HKG list generated by ourselves according to normal tissue data (GSE3526 and GSE7307) [27]. The overlap with the DPG list is 198, 197, 307, and 214 genes (10.7%, 11.4%, 10.8%, and 11.8% in the corresponding HKG list, respectively; the four datasets include what have been reported by She et al, Tu et al, Zhu et al, and the current study). The intersection and the union are 5 and 600 genes, respectively, and the result indicates that only 5 of the 1,728 DPGs are commonly accepted as HKGs and 600 DPGs are potential HKGs. Interestingly, among the 600 genes, 244 are organized as 122 DPG pairs. This is a rather high tendency for house-keeping DPGs to cluster together although the fraction of HKGs in DPGs are close to random selection (1/3 of the human total genes are empirically defined as potentially house-keeping). A more detailed functional analysis is shown in **Figure 5**. The pie chart is generated by using the Cytoscape plugin “MultiColoredNodesPlugin” [28]. The large portion of overlap between DPGs and HKGs include energy metabolism and RNA processing; module 2 is the major cluster formed by both DPGs and HKGs. Overall, although DPGs and HKGs are both involved in the core cellular functions and conserved during evolution, the fraction of house-keeping DPGs are restricted and clustered in a subset of functional categories among all DPG functions.

### Tissue-specific expression of DPGs

To further characterize tissue-specific functions of DPGs, we examined the expression pattern of DPGs among different tissues. The major microarray dataset used in this study is GSE3526, which contains 20 CNS tissues and 45 non-CNS tissues. This dataset was chosen because of the wide coverage of different tissue types within one dataset for a fair comparison. Within the 20 CNS tissues, we used cerebellum as control and other 19 CNS tissues as cases. For the 45 non-CNS tissues, we used skeletal muscle as control and other 44 tissues as cases. We also compared the CNS tissues with skeletal muscle or all of the non-CNS tissues, and compared cerebellum with skeletal muscle to reveal the difference. The selection of controls was based on the hierarchical clustering result by Roth et al [27]. Two types of between-tissue differences were examined: an absolute pathway score to survey the different expression of DPGs between tissues and a relative pathway score to evaluate the different expression changes between DPGs and other genes in a pair of tissues (detailed in Methods).

Among the CNS tissues, the variance of gene expression is mostly small in modules 1 and 2, and larger variance is seen mostly in the functional sets outside of the two main modules (**Figure S4**). The most significant difference is found in corpus callosum, medulla, and spinal cord, all of which are at the connection zone between CNS and peripheral tissues (data not shown). For non-CNS tissues, much higher between-tissue difference is found (**Figure S5**). However, module 1 remains as the least variable, indicating the fundamental role of chromosome maintenance in all the tissues. The most variable functional sets among non-CNS tissues include energy metabolism and cancer signaling gene sets, neither is significantly variable among CNS tissues, suggesting the critical role of energy homeostasis and tight control of cell proliferation in CNS tissues [29]. Additionally, the cross comparison between CNS and non-CNS tissues further reveals network-wide differences between the two groups of tissues (data not shown).

### Dynamic perturbation of DPGs in the course of HIV infection

In order to further unveil the roles of DPGs in disease mechanism, we examined the differential expression pattern of DPGs in several diseases including pathogen infections (HIV, malaria, lymphoma, tuberculosis, ASLE, and Streptococcus infection) and cancers (ATL, hepatocellular carcinoma, kidney cancer, lung cancer, and sporadic colorectal cancer). We found that DPGs in cancers display much higher differential expression than that in infection diseases (data not shown). More importantly, we found an interesting dysregulation pattern of DPGs in HIV. The main findings based on the absolute pathway score are as follows. First, significant differential expression is found in both CD4+ and CD8+ T cells when comparing the acute and chronic stages with the negative controls. The observed change of gene expression may reflect the action of the host defense mechanism. Second, there are no significant differences between early infection and chronic stage in CD4+ T cell, which are attributable to the early established HIV-1 infection [30]. Third, there are no significant differences between long-term nonprogressor patient and uninfected controls. Fourth, CD8+ T cells have much more differentially expressed genes (DEGs) and higher level of differential expression than CD4+ T cells, which is interpreted as a possible artifact of microarray analysis methods, or higher heterogeneity in CD4+ T cells.

Distinct contribution of DPGs can be revealed based on the relative pathway score and the changes of perturbation during infection process can be observed (**Figure S6 and S7**). Although there is no significant difference on DEGs between the acute and chronic stages, DPGs seem to play more active role in many functional categories. First, DPGs display no significant difference from non-DPGs in module 2 for both CD4+ and CD8+ T cells, indicating coordinated perturbations of all the genes (both DPGs and non-DPGs) in module 2. Second, DPGs display higher perturbation relative to non-DPGs at the acute stage in CD8+ T cells, while they display higher perturbation at the chronic stage in CD4+ T cells. This different perturbation pattern of DPGs between CD4+ and CD8+ T cell is likely due to their different roles in the immune response. Almeida et al suggested that CD8+ T cells function as the superior control of DNA replication of HIV-1 virus in CD4+ T cells [31].

**Figure 6:**
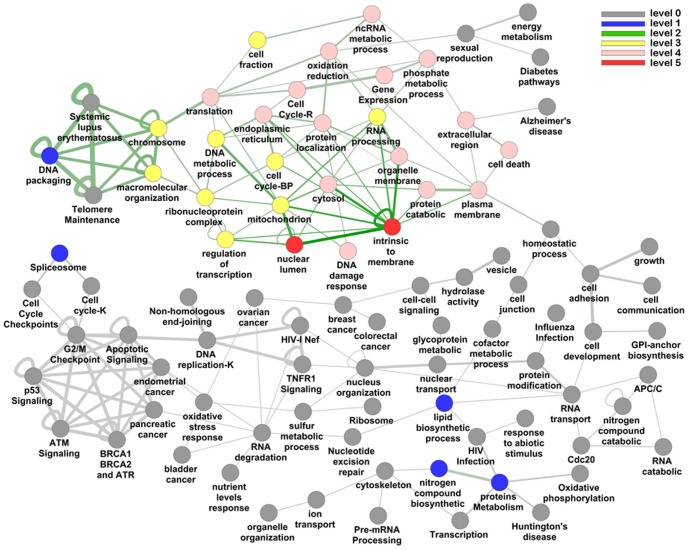
The perturbation pattern of DPGs in HIV elite controllers based on the dataset GSE23879. The relative pathway scores are used and the different colors represent different perturbation levels.

For long-term non-progressors, we found that DPGs show higher perturbation than non-DPGs. Similar situation is found in the comparison of HIV-1 elite controllers and negative controls from the dataset GSE23879. In contrast to long-term non-progressors, elite controllers maintain the level of HIV-1 replication that is undetectable by standard commercial assays and do not have acute and chronic stages [32], [33]. For both elite controllers and long-term non-progressors, DPGs with the functions of “chromosome”, “cell death” and “gene expression” present higher perturbation than non-DPGs. In addition, high relative scores in “RNA processing”, “energy metabolism”, “cell cycle”, especially “nuclear lumen” and “intrinsic to membrane” are observed in elite controllers. It is evident that every functional set in module 2 displays difference while very few functional sets in modules 3 and 4 show any differences (**Figure 6**). This suggests that perturbation of DPGs in module 2 may be linked to the HIV resistance mechanism.

## Conclusion

In this work, we have conducted comprehensive analysis on the functional connection of DPGs that are special form of gene organization and cover a broad range of functions including core cellular and environmental response related functions. An interesting finding is that DPG pairs tend to be partitioned into different pathways and thus enable simultaneous control or connecting of two or more pathways by a single promoter. Most CNS tissues have similar DPG expression patterns, while the patterns are more diverse for non-CNS tissues. Based on detailed evaluations, we suggest that DPGs may contribute to HIV resistance mechanisms, and based on their overall conservation across species and lineages and involvement in diverse functional networks, we propose that DPGs may be a class of genes evolved to create coordination for both conserved core house-keeping and tissue-specific functions.

## Methods

### DPG information

The human DPG information was obtained from LCGbase [16]. We also used gene annotations from both NCBI (ftp://ftp.ncbi.nlm.nih.gov/) and HGNC databases (http://www.genenames.org)[34] and gene ID conversion tool from DAVID (http://david.abcc.ncifcrf.gov/conversion.jsp). The dataset contains 864 DPGs.

### GO similarity

The evaluate of GO similarity was based on the R package GOSemSim, which implements four information content (IC)- and a graph-based methods, and made the comparison among the methods easily [35]. The Schlicker's methods were finally used for calculating GO similarity score, as it corrected the “shallow annotation problem” [36], [37]. To acquire comprehensive relationships among DPGs, we considered the IEA evidence code in GO terms [37] and used the method of “rcmax.avg” in GOSemSim to combine semantic similarity scores of multiple GO terms. We also randomly sampled 100,000 gene pairs as background from genes with consistent Ensembl ID and record in LCGbase.

### Network construction

We clustered 1,728 (864 paris) human DPGs into functional categories based on the Biochart analysis by DAVID [38], with annotations from disease (genetic_association_db_disease [39]), pathway (Reactome [40], biocarta http://www.biocarta.com/, and KEGG or Kyoto Encyclopedia of Genes and Genomes http://www.genome.jp/kegg/ [41]), and Gene Ontology (BP, CC) [42]. We used category size >5 and overlap <80% as criteria to filter the chart results and obtained 114 representative categories which contained 1,299 human DPGs (total coverage 75.23%; since only 1,359 DPGs were annotated in these datasets, the relative coverage was 95.58%). Therefore, these nodes basically covered all the functions of DPGs. Of all the genes in these categories, DPGs often constituted 10∼20% of each category (average 22.3% for KEGG, 20.5% for Reactome, 23.54% for Biocarta, 13.78% for Disease, 11.96% for GO BP, and 10.79% for GO CC). Three representative categories were non-homologous end-joining in KEGG (46.15%, 6 of 13 genes), telomere maintenance in Reactome (37.5%, 21 of 56 genes), and RNA polymerase in KEGG (35.7%, 10 of 28 genes) (more details in **Table S2**).

In order to reproducibly build functional network connected by DPGs, we first constructed two network maps: an overlap map and an interaction map. Since most genes were annotated by more than one term, overlaps were common among the functional gene sets and were displayed in the overlap map. Similarly, DPG pairs tended to be separated into two related pathways, so they can be organized by the interaction map. When the two types of maps led to different network topologies, only the interaction map was kept. The interaction rate >0.07 was chosen as the cutoff to retain as many functional sets as possible (90 functional sets, 1,262 DPGs, relative coverage of 92.86%). The modules on the network were automatically generated by the Cytoscape plugin “ClusterONE”[23] and the parameters of “Multi-pass” and “Simpson coefficient” were used for our analyses.

### Microarray data collection

Datasets for gene expression profiling were downloaded from GEO database (http://www.ncbi.nlm.nih.gov/gds). We retrieved datasets for normal tissue (GSE3526) [27], aging (GSE16487) [43], and various diseases, which included HIV: GSE6740, GSE9927, GSE18233, GSE23879; malaria: GSE5418; tuberculosis: GSE19491; ATL (adult T-cell leukemia/lymphoma): GSE14317; ASLE (human active and latent tuberculosis): GSE19491; Streptococcus infection: GSE19491; multiple cancer: GSE2109; breast cancer: GSE27562; hepatocellular carcinoma: GSE14520; kidney cancer: GSE15641; lung cancer: GSE18842; and sporadic colorectal cancer: GSE23878) [17], [30], [33], [44], [45], [46], [47], [48], [49], [50], [51], [52].

### Absolute and relative pathway scores

We used two measures to reflect the perturbation of the 90 functional categories under different conditions: an absolute pathway score and a relative pathway score. The absolute score was focused on differential expression of DPGs in each functional set. We calculated p value of differential expression for every gene between the case and control groups using an R package Limma [53]. The control for CNS tissues was cerebellum and for non-CNS it was skeletal muscle, the selection of which was based on the hierarchical clustering result by Roth RB et al. [27]. We converted this p value to a score.
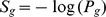



The pathway score was the average of quartiles (Q1, Q2 and Q3) of DPG genes in a pathway.
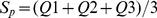



We defined the categories with the score less than 1.301 as no perturbation, corresponding to the –log(p value = 0.05), while scores at 1.301–2, 2–3, 3–4, 4–6, and 6– were assigned to level 1 to 5 differential expressions, corresponding to the p value of 0.05–0.01, 0.01–1E-3, 1E-3–1E-4, 1E-4–1E-6, and 1E-6–, respectively.

The relative score was focused on the differential perturbation patterns between DPGs and all genes, which reflected the difference between DPGs and non-DPGs. The details were similar to previous study [54], and ten thousand random permutations were performed.
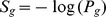


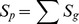



The significant of S_p_ depended on the frequency of comparing with randomly sampled genes. Then we calculated a score for every category (score  =  frequency/permutation), which was further converted to level 0 to 5 differential expressions (score <0.5, 0.5–0.6, 0.6–0.7, 0.7–0.8, 0.8–0.9, and 0.9–1).

## Supporting Information

Figure S1 The overlap pattern of 114 functional DPG sets. Only overlap rate >30% is included to leave the layout cleaner. The edge color depends on the number of overlaps between sets and the edge width on the overlap rate between sets. The overlap and interaction patterns are both considered when laying out these 114 sets. The linkages with both higher overlap and interaction rates are circled in red.
(TIF)

Figure S2 The interaction pattern of 114 functional DPG sets. Only interaction rate >7% is included to make the layout cleaner. The edge color depends on the number of interacting gene pairs between the sets and the edge width on the interaction rate between the sets. The overlap and interaction pattern are both considered when laying out the 114 sets. The linkages with both higher overlap and interaction rates are circled in red.
(TIF)

Figure S3 The distribution of top 10 correlated functional sets. Different node colors are used to distinguish the correlation, and the nodes with same color indicate higher correlation.
(TIF)

Figure S4 Variance of DPG expression among CNS tissues. Different colors represent different range of variance (green for 0.4–0.5, yellow for 0.5–0.7, red for >0.7).
(TIF)

Figure S5 Variance of DPG expression among non-CNS tissues. Different colors represent different range of variance (green for 0.4–0.5, yellow for 0.5–0.7, red for >0.7).
(TIF)

Figure S6 The change of perturbation pattern of functional set during HIV process in CD4 T cell. The relative pathway score and perturbation level are used. The different colors represent the change direction of the set; the color deepness is related to the intensity of the change. The infection process includes acute stage, chronic stage and long-term nonprogressor stage.
(TIF)

Figure S7 The change of perturbation pattern during the HIV infection process in CD8+ T cell. The relative pathway score is used. Different colors represent different patterns; the color deepness is related to the intensity of the change. The infection processes include acute, chronic, and long-term nonprogressor stages.
(TIF)

Table S1 Information on Human DPGs.
(XLS)

Table S2 Detailed information on the functional categories of gene networks.
(XLS)
