# Supplementary figures and images for: Functional Networking of Human Divergently Paired Genes (DPGs)
Source: PLoS One. 2013 Oct 31;8(10):e78896. doi: 10.1371/journal.pone.0078896 (PMC3815023; doi:10.1371/journal.pone.0078896)

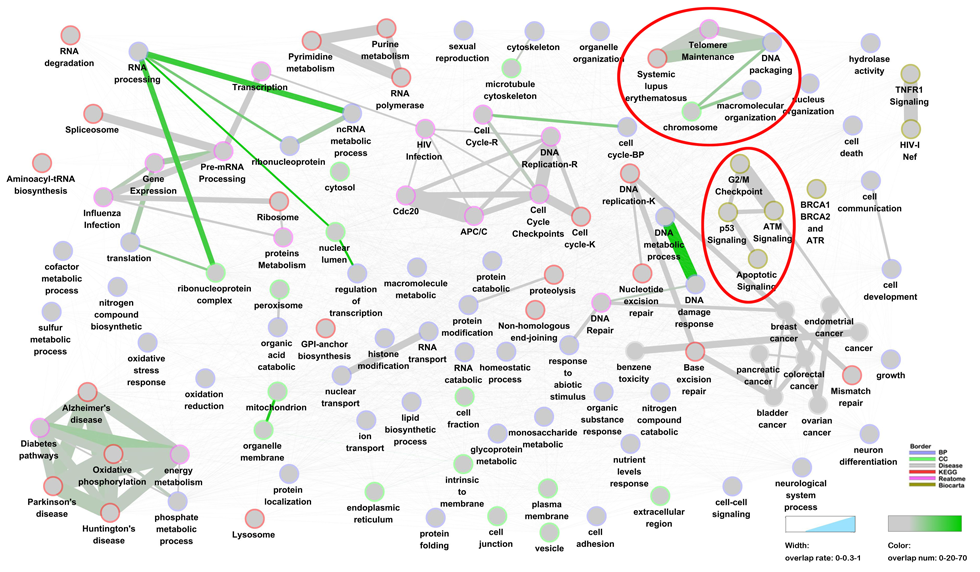

Supplement: Figure S1 — The overlap pattern of 114 functional DPG sets. Only overlap rate >30% is included to leave the layout cleaner. The edge color depends on the number of overlaps between sets and the edge width on the overlap rate between sets. The overlap and interaction patterns are both considered when laying out these 114 sets. The linkages with both higher overlap and interaction rates are circled in red. (TIF) [file pone.0078896.s001.tif]

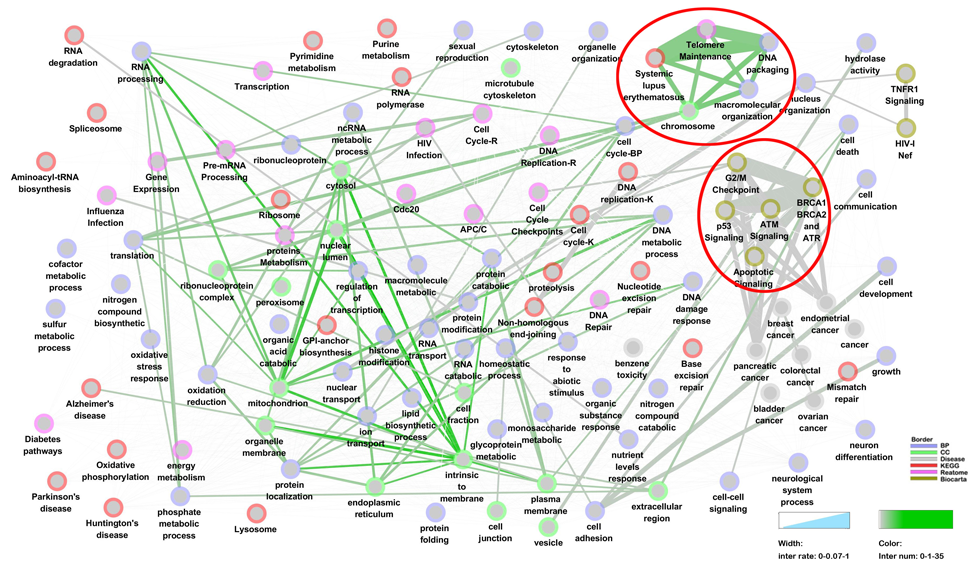

Supplement: Figure S2 — The interaction pattern of 114 functional DPG sets. Only interaction rate >7% is included to make the layout cleaner. The edge color depends on the number of interacting gene pairs between the sets and the edge width on the interaction rate between the sets. The overlap and interaction pattern are both considered when laying out the 114 sets. The linkages with both higher overlap and interaction rates are circled in red. (TIF) [file pone.0078896.s002.tif]

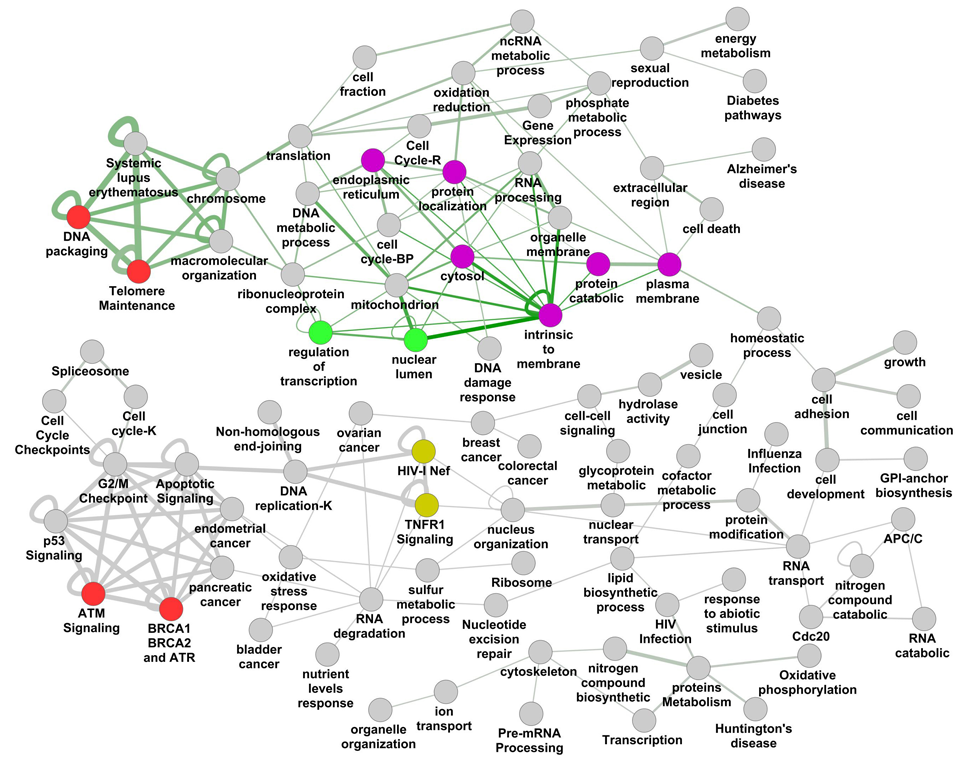

Supplement: Figure S3 — The distribution of top 10 correlated functional sets. Different node colors are used to distinguish the correlation, and the nodes with same color indicate higher correlation. (TIF) [file pone.0078896.s003.tif]

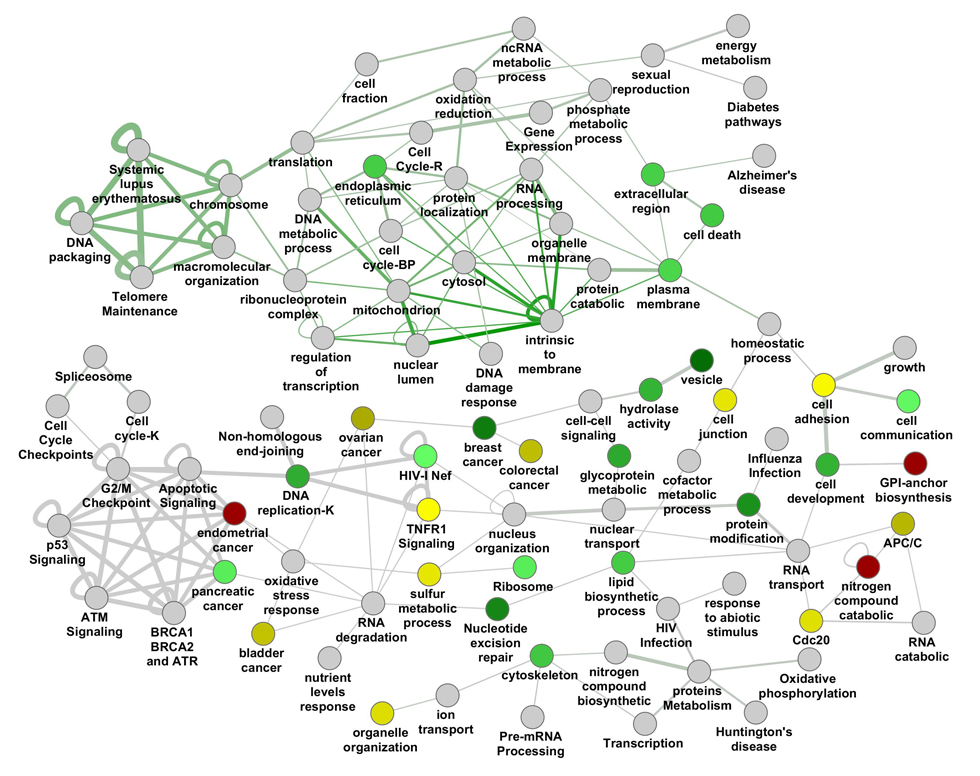

Supplement: Figure S4 — Variance of DPG expression among CNS tissues. Different colors represent different range of variance (green for 0.4–0.5, yellow for 0.5–0.7, red for >0.7). (TIF) [file pone.0078896.s004.tif]

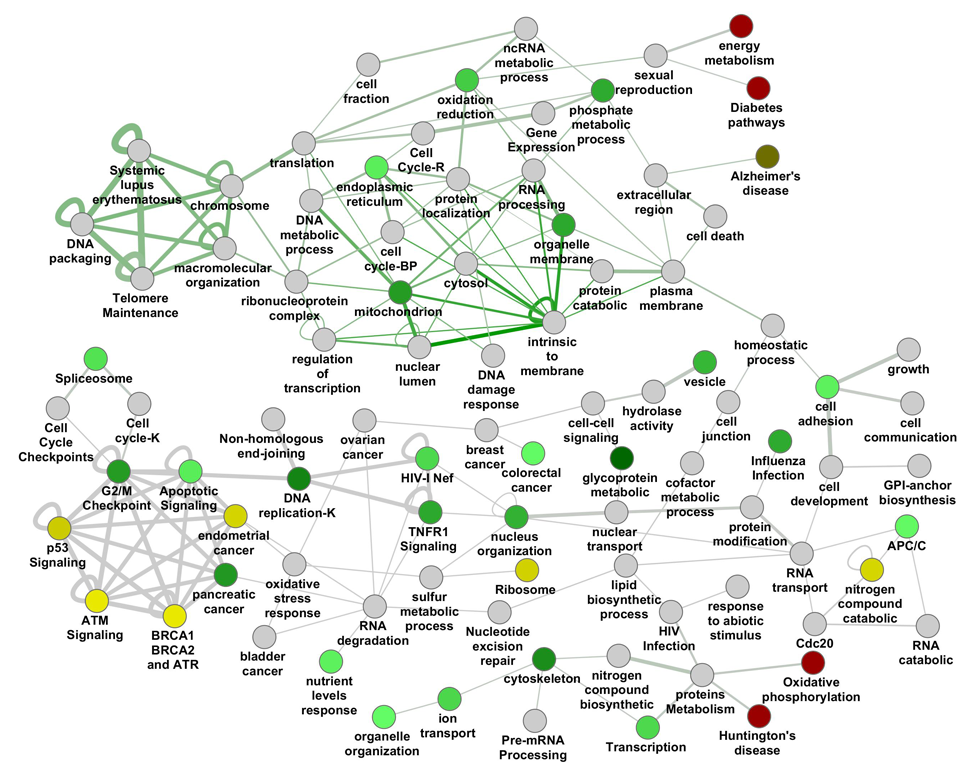

Supplement: Figure S5 — Variance of DPG expression among non-CNS tissues. Different colors represent different range of variance (green for 0.4–0.5, yellow for 0.5–0.7, red for >0.7). (TIF) [file pone.0078896.s005.tif]

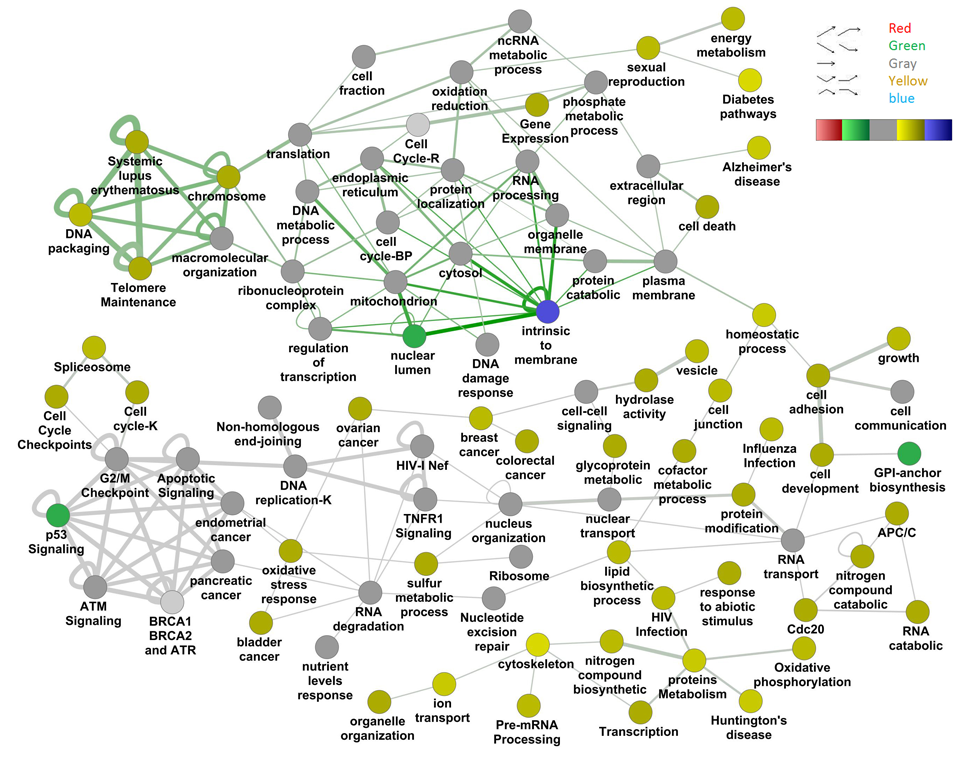

Supplement: Figure S6 — The change of perturbation pattern of functional set during HIV process in CD4 T cell. The relative pathway score and perturbation level are used. The different colors represent the change direction of the set; the color deepness is related to the intensity of the change. The infection process includes acute stage, chronic stage and long-term nonprogressor stage. (TIF) [file pone.0078896.s006.tif]

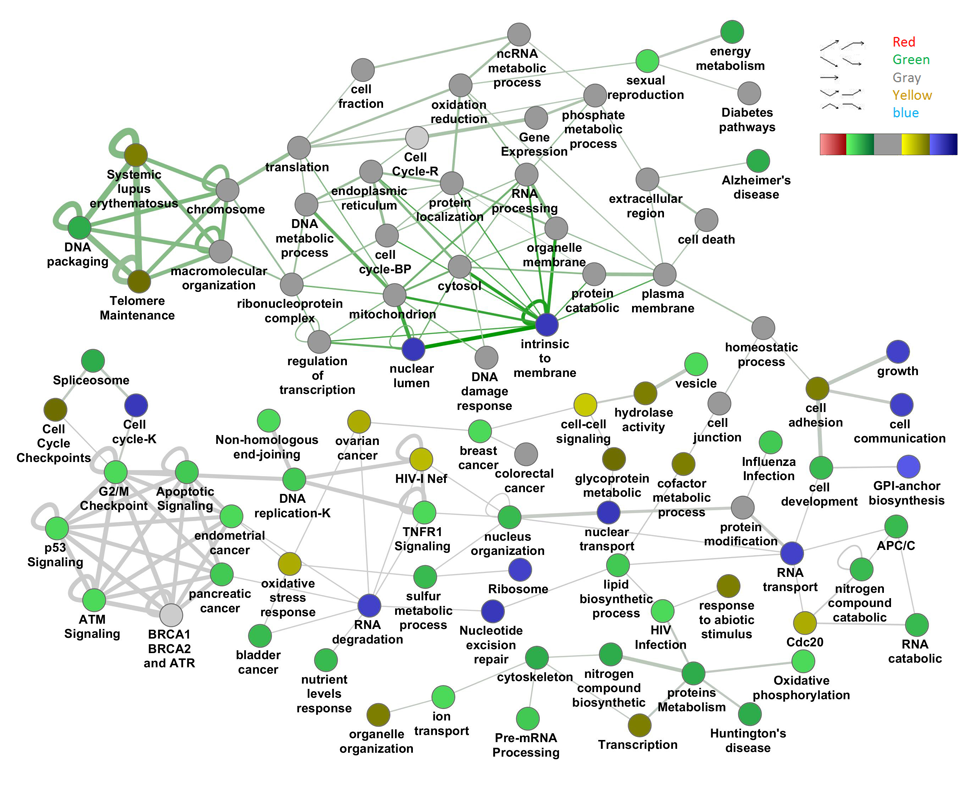

Supplement: Figure S7 — The change of perturbation pattern during the HIV infection process in CD8+ T cell. The relative pathway score is used. Different colors represent different patterns; the color deepness is related to the intensity of the change. The infection processes include acute, chronic, and long-term nonprogressor stages. (TIF) [file pone.0078896.s007.tif]
